# Impact of Image Content on Medical Crowdfunding Success: A Machine Learning Approach

**DOI:** 10.2196/58617

**Published:** 2024-11-15

**Authors:** Renwu Wang, Huimin Xu, Xupin Zhang

**Affiliations:** 1 School of Economics and Management East China Normal University Shanghai China

**Keywords:** medical crowdfunding, visual analytics, machine learning, image content, crowdfunding success

## Abstract

**Background:**

As crowdfunding sites proliferate, visual content often serves as the initial bridge connecting a project to its potential backers, underscoring the importance of image selection in effectively engaging an audience.

**Objective:**

This paper aims to explore the relationship between images and crowdfunding success in cancer-related crowdfunding projects.

**Methods:**

We used the Alibaba Cloud platform to detect individual features in images. In addition, we used the Recognize Anything Model to label images and obtain content tags. Furthermore, the discourse atomic topic model was used to generate image topics. After obtaining the image features and image content topics, we built regression models to investigate the factors that influence the results of crowdfunding success.

**Results:**

Images with a higher proportion of young people (β=0.0753; *P*<.001), a larger number of people (β=0.00822; *P*<.001), and a larger proportion of smiling faces (β=0.0446; *P*<.001) had a higher success rate. Image content related to good things and patient health also contributed to crowdfunding success (β=0.082, *P*<.001; and β=0.036, *P*<.001, respectively). In addition, the interaction between image topics and image characteristics had a significant effect on the final fundraising outcome. For example, when smiling faces are considered in conjunction with the image topics, using more smiling faces in the *rest and play* theme increased the amount of money raised (β=0.0152; *P*<.001). We also examined causality through a counterfactual analysis, which confirmed the influence of the variables on crowdfunding success, consistent with the results of our regression models.

**Conclusions:**

In the realm of web-based medical crowdfunding, the importance of uploaded images cannot be overstated. Image characteristics, including the number of people depicted and the presence of youth, significantly improve fundraising results. In addition, the thematic choice of images in cancer crowdfunding efforts has a profound impact. Images that evoke beauty and resonate with health issues are more likely to result in increased donations. However, it is critical to recognize that reinforcing character traits in images of different themes has different effects on the success of crowdfunding campaigns.

## Introduction

### Background

Charitable crowdfunding has revolutionized philanthropy and social support, providing a unique and accessible platform for individuals and organizations to raise funds for their causes. In the realm of medical crowdfunding, patients often turn to web-based platforms such as GoFundMe for financial support.

A critical element of a successful crowdfunding campaign is the selection of an effective campaign cover image. This image serves as a visual representation of the project and plays a key role in attracting potential donors [1-3]. It also helps to communicate the project’s core information and value proposition, allowing potential supporters to quickly understand the project’s goals and importance of the project [4,5].

In the current digital age, the campaign cover image serves as a first impression. Given people’s limited attention span, it has a significant impact on the outcome of the crowdfunding campaign [6,7]. Research has shown that individuals process and retain visual information better than textual data [8-12]. The importance of visual information has been demonstrated in advertising, marketing, technology crowdfunding, and other fields [13-16]. Researchers have investigated several factors that influence the effectiveness of crowdfunding campaigns, such as project descriptions, incentives, and engagement on social media platforms [17-21]. While there is growing public awareness of using web-based crowdfunding platforms to raise medical funds for patients with cancer, there is a lack of research in academia on how specific elements of a cancer crowdfunding project’s image affect its success rates.

In order to obtain a more profound understanding of the influence of images on the success of cancer crowdfunding campaigns, we performed a study of cancer-related crowdfunding campaigns on GoFundMe, which is the most extensive site for medical crowdfunding. Our hypotheses are shown in Textbox 1.

**Our hypotheses.**

- Individual characteristics of images used for medical crowdfunding projects significantly influence the projects’ fundraising outcomes.
- The thematic content of images is a significant determinant of the success of crowdfunding campaigns.
- There is a significant interaction effect between the subject matter of images and individual characteristics of crowdfunding images, which in turn affects the funding results of medical crowdfunding projects.

### Related Work

#### Individual Characteristics of Crowdfunding Images

Scholars have confirmed the importance of character appearance and character attributes in images, including the number of faces, facial expressions, race, and gender. Some researchers have shown that the presence of faces greatly facilitates funders’ decision-making [22-24], but there are also areas of research that demonstrate that the disclosure of a project initiator’s real face negatively impacts crowdfunding success [25]. In addition, studies suggest that both positive expressions, including smiling, as well as negative expressions, such as sadness, can have an influence [26-28].

Compared to other types of crowdfunding projects, medical crowdfunding for cancer projects has its own specific focus and target audience. Therefore, factors that have been shown to positively or negatively influence people’s success in crowdfunding in other fields [29,30] may not be applicable to cancer projects in medical crowdfunding.

#### The Topic of Image Content

While empirical studies of crowdfunding data have increased, there is still a lack of attention to image content, highlighting the need for broader adoption of visual data analysis in research. Some academic discourse has focused on the photographic aspects of images, such as color dynamics, composition, and figure-ground relationships [31,32]. However, for crowdfunding initiators, launching a crowdfunding project and uploading images that take into account photographic features require a certain technical threshold. The content of the image is more easily understood and judged by the general public, which can be an important factor in determining whether it is suitable for uploading. Platforms such as Indiegogo emphasize the importance of an image’s style and content as critical factors for achieving crowdfunding success. Therefore, when evaluating the impact of visual elements, it is imperative to place significant emphasis on the role of image content in crowdfunding results.

Previous research has analyzed imagery used by nonprofit organizations in fundraising appeals and raised concerns about the possibility of perpetuating harmful stereotypes. The depiction of individuals as passive, impoverished, and victimized, waiting for rescue by the Western spectatorship, has been examined for its potential to reinforce racial and economic clichés [33,34]. Careful selection of image content is crucial, as potential donors rely solely on the information presented within a crowdfunding campaign due to their limited firsthand experience. The content of images plays a pivotal role in shaping their initial perceptions of individuals in need of aid.

Research has shown that image content significantly influences crowdfunding campaign donations from all perspectives. For example, images with self-help behavior content attract more donations [35]; the presence of a direct gaze action in campaign images leads to higher fundraising results [36]; and images depicting a patient’s current serious medical condition are more effective than images depicting a relatively normal image of that condition [37].

Images with varying content have distinct persuasive effects when used in the domain of health care crowdfunding [24]. Emotional and credibility-based images display a positive persuasive effect, while logos-related images depict a negative persuasive effect.

#### The Intersection of the Factors in Crowdfunding

The convergence of factors in crowdfunding projects is influenced by a variety of elements, and the effects of the interplay between these factors on the final crowdfunding outcome are underresearched.

Previous studies have attempted to investigate the relationships between visual and textual factors. Zhao et al [38] addressed potential interactions between visual emotions and textual emotions that had previously been ignored. Their study showed that emotional congruence between images and linguistic approach can mitigate the outcomes of unsuccessful web-based charity crowdfunding campaigns. Yang et al [16] investigated how the number of images and videos affects the relationship between text length and crowdfunding outcomes. They observed a positive correlation between text length and fundraising outcomes; however, this relationship weakened as the number of videos and images increased. Buskila and Perez [35] attempted to analyze the various features of images, focusing on whether or not the characters in the images were rescued, as well as the emotions conveyed by the images. The study concluded that victims received fewer donations due to 2 specific factors, including the use of images depicting negative emotions and the absence of self-help behavioral content.

However, due to limited research, scholars are unable to gain a deeper understanding of the interactions between the elements in the image. Based on the above research, this paper conducts further research on the interactions between various elements in crowdfunding programs.

## Methods

### Overview

**Figure 1:**
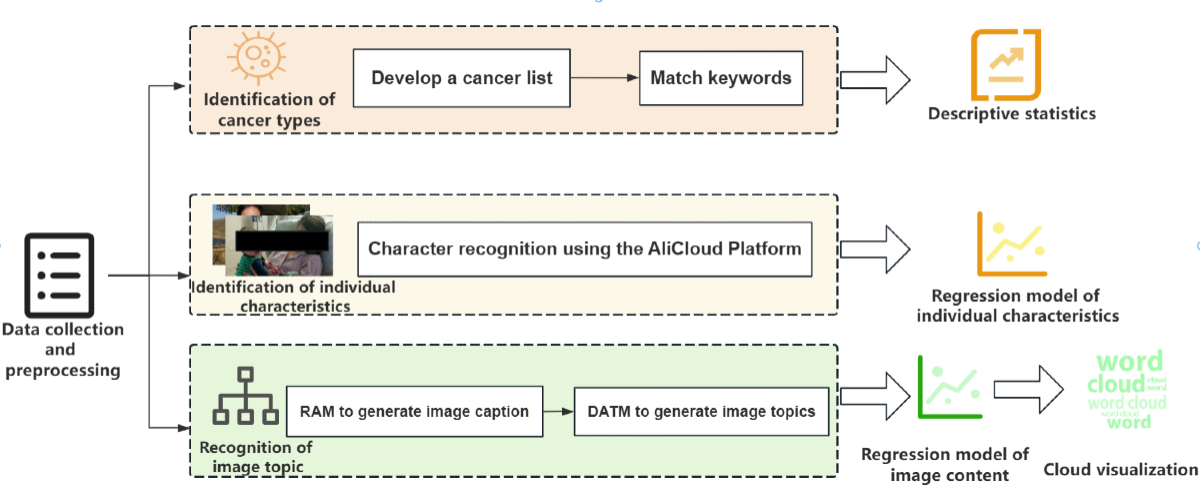
Technological route throughout the paper. AliCloud: Alibaba Cloud; DATM: discourse atomic topic modeling; RAM: Recognize Anything Model.

As shown in Figure 1, this section describes the flow of the research throughout the article in 5 parts.

### Data Collection

We used a Python-based web-scraping program to collect data on all cancer-related crowdfunding projects hosted on the platform from January 1, 2019, to July 28, 2020. Ultimately, our efforts yielded data from a total of 307,886 projects available on the site. The dataset includes essential project details such as basic information, textual content, and images. In addition, the basic information includes several key variables that are critical for later analysis, including funding amount, number of donations, description length, title length, and duration. These elements are referred to as basic features.

Given that the United States is a major player in the global crowdfunding market, with a well-established environment and individuals often demonstrating a higher level of engagement and trust in crowdfunding efforts, we focused our initial screening on US-based projects with individual crowdfunding initiators. This refinement narrowed our dataset to 243,795 medical campaigns. We then transformed the dataset to facilitate analysis. This involved converting the fundraising amounts from textual to numerical values and calculating the time elapsed since the creation of each campaign using a predetermined base date.

### Procedure

#### Identification of Cancer Types

Different types of cancer have significant variations in disease etiology, treatment methods, and costs [39]. These differences can have a significant impact on the goals and outcomes of crowdfunding initiatives [40]. Therefore, it is essential to identify the specific cancer type associated with each crowdfunding project and analyze its characteristics using different cancer classifications. Crowdfunding initiators usually provide detailed information in the project description, including the relevant cancer type details. Therefore, identifying the cancer type through the description is crucial. We compiled a list of 238 cancer categories and their corresponding keywords to perform a keyword matching analysis of crowdfunding project descriptions. In the end, we identified 97 different cancer types after analyzing a total of 243,795 crowdfunding projects (Table 1).

**Table 1:** Top 10 cancers in terms of the number of projects.

Cancer type	Projects, n
Skin cancer	40,038
Childhood cancer	16,714
Brain tumors	7251
Lung cancer	6495
Breast cancer	6338
Kidney cancer	6185
Familial gastrointestinal stromal tumors	5482
Liver cancer	5237
Gastric cancer	4990
Eye cancer	4978

#### Identification of Image Features

In crowdfunding cover images, individuals can provide supplementary information about patients and other project aspects, allowing potential donors to better comprehend and empathize with the project, ultimately increasing support likelihood. This study used the face attribute recognition interface provided by Alibaba Cloud’s Vision Intelligent Application Programming Interface Platform [41] to detect personal features in crowdfunding project images. The face detection and recognition algorithms of this platform can realize high-performance attribute recognition.

We conducted analysis of the uploaded crowdfunding project images by designing a Python program to interface with the application programming interface. The results returned by the interface included *Face Count*, *Male Count*, *Female Count*, *Age*, *Smile Count*, *Glasses Count*, and *Hat Count* for each image, as shown in the Table 2.

**Table 2:** Examples of image features in crowdfunding images^a^.

Picture	Face Count, n	Male Count, n	Female Count, n	Smile Count, n	Glasses Count, n	Hat Count, n	Age (years)
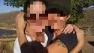	2	0	2	2	0	0	36-60 and 60
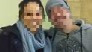	2	1	1	2	0	1	18-35

^a^The people in the pictures are mosaicked to protect privacy.

#### Image Caption on the Recognize Anything Model

In addition to exploring the impact of image features in crowdfunding images, the cancer crowdfunding project’s image content can provide additional insights into the project. To characterize the content of these images, we used the Recognize Anything Model (RAM) [42].

The RAM integrates the Segment Anything scene segmentation model from Meta [43] with the recognition capabilities of Zero Shot. This combination allows the RAM to localize and identify object categories within images. It recognizes over 6400 common labels, encompassing a broader range of pertinent categories compared to Open Images V6 [44]. More details about the RAM can be found in Multimedia Appendix 1.

Through configuring local parameters, we standardized and resized images. We then used the *ram-swin-large_14m* pretrained model to process images from cancer crowdfunding projects, generating results using the RAM.

#### Discourse Atomic Topic Modeling

However, the complexity of image descriptions makes them unsuitable for subsequent impact analysis on crowdfunding results. Therefore, we performed topic recognition on image descriptions to uncover commonalities hidden within the descriptive content. We then used this comprehensive integration of image content for further analysis.

In the process of recognizing image themes, we used the discourse atomic topic modeling (DATM) technique [45]. This method consists of 3 basic steps. First, the data are distilled into the most salient features. Second, we used latent variable modeling to detect potential themes in the corpus. Finally, we used sentence embedding to tightly link these themes to the specific contexts observed.

In order to reveal the topics within this embedding space, we applied K-singular value decomposition, a sparse dictionary learning algorithm introduced by Aharon et al [46] in 2006, to the word vectors. This algorithm yields a set of K vectors called “discourse atoms.” Each of the V word vectors in the vocabulary can be expressed as a sparse linear combination of these atom vectors. Using the generative model outlined below (equation 1), the atom vectors are constructed as topics within the embedding space.



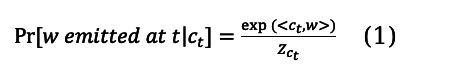



Equation 1 is also a simplified version of the latent variable model. The formula expresses the probability of a word w being present at some location *t* in the corpus. *c_t_* is a vector in the semantic space that represents the underlying meaning of the word in the current context. The similarity between its word vector w and the latent “gist” at that point in the corpus *c_t_* is computed. *Z_ct_* is the sum of the similarities so that the distribution sums to 1.

The formula expresses the probability of a word w being present at some location *t* in the corpus. We used smooth inverse frequency embeddings to derive themes from the observed phrases. Smooth inverse frequency embeddings are calculated using the maximum a posteriori estimate of the combined contextual vectors for a set of contextual terms.



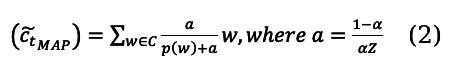




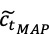
 is a weighted average of the word vectors in the context window; words are weighted based on their corpus frequency *p*(*w*). Frequent words make a smaller contribution to the estimate of 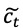. The α is a hyperparameter.

This procedure allows us to discover latent topics in a corpus and identify the topic that best matches the estimated gist of an observed context window. Finally, we used the relevant words to name each topic, resulting in corresponding topic types for each image.

Since image descriptions frequently comprise many insignificant articles and prepositions, the corpus used for clustering image topics relies on the image tags generated by the RAM. We identified the optimal combination of parameters by evaluating different combinations using root mean squared error. The best results were obtained with a configuration of 7 topics, each with a maximum of 2 atoms per topic, meaning each term could be used in up to 2 topics.

#### Evaluation of Topic Model Results

Evaluation methods are necessary for assessing the image descriptions recognized by the RAM and the results of topic discovery of image descriptions by DATM.

Topic coherence is a measure of the relatedness and interpretability between words within a topic. The topic coherence proposed by Newman et al [47] uses Pairwise Pointwise Mutual Information for the coherence between topic words. Coherence scores are computed by taking into account the co-occurrence of words in a given context (eg, a document or a sliding window), with the goal of evaluating whether words within a topic are “coherent,” that is, whether they form meaningful associations. In our study, we selected the topics with the highest topic coherence from the generated topics by varying the number of the topics and the number of atoms.

In addition, topic diversity [48] is an indicator of variation among topics. The way to find the result for topic diversity is to determine the percentage of representative words of the current topic that do not appear in the representative words of other topics.

### Variables

By collecting data and identifying project features, we had gathered specific variables that could potentially affect cancer crowdfunding projects. These variables consist of project basic information, cancer types, image features in crowdfunding images, and the topic of image content.

*Funding Percentage* is a continuous variable, indicating the percentage of the target amount that the current project has raised. We used *Funding Percentage* to measure the outcome of a crowdfunding project.

In the process of selecting the independent variables, we considered a combination of interdisciplinary literature [22-27] that identifies individual characteristics such as the number of faces, gender, and facial expression. While these factors have been empirically supported in other domains, their effects have yet to be validated in the specific context of health care crowdfunding images. Furthermore, given the centrality of image content in the operational strategies of crowdfunding platforms such as Indiegogo, the visual presentation of different thematic content has been shown to significantly modulate crowdfunding outcomes [35-37]. Therefore, the inclusion of “topic” as an independent variable aims to analyze how it plays a unique role in the field of health care crowdfunding (Table 3).

**Table 3:** Description of the variables of the regression model, including dependent variable and image characteristics.

Variable	Description
Funding Percentage	The ratio of the donation amount to the target amount
Funding Amount	The amount of donations received
Donation Count	The number of donations received
Description Length	The length of the project description for the crowdfunding project
Title Length	The length of the title description of the crowdfunding project
Time	The time interval between the creation of the crowdfunding project and the crawling of the data
Face Count	Number of individuals in each image
Female Percent	Percentage of female individuals in each image
Age <18	Percentage of individuals younger than the age of 18 years in each image
Age 18-35	Percentage of individuals aged 18-35 years in each image
Age 36-60	Percentage of individuals aged 36-60 years in each image
Age ≥60	Percentage of individuals aged over 60 years in each image
If Smile	Whether there is a smiling face in each image
Topic	The topic of image caption

### Regression Model

In order to investigate the influence of image characteristics on crowdfunding results, a logistic regression model was used. In this study, regression models were built to predict crowdfunding outcomes in the presence of multicollinearity as determined by the variance inflation factor value. Additionally, the regression results were visualized as a word cloud to visually illustrate the content of different image themes and their effects by using Python’s *WordCloud* package.

### Ethical Considerations

The data sources for this study were information available on public platforms, and data collection did not involve direct participation of study participants. To protect privacy, all data were anonymized to ensure that individuals could not be identified from the results. Additionally, all data were stored on a secure server with access restricted to project team members, in accordance with a strict privacy policy.

## Results

### The Impact of Image Features in Crowdfunding Images on Crowdfunding Outcomes

Of the 243,795 images, 205,496 contained depictions of human figures, indicating a *Face Count* ≥1. The dataset was largely dominated by single-person images, accounting for 58.83% (120,893/205,496) of the total. Multiperson images included from 2 to 10 individuals. Regarding gender distribution, 42.31% (86,945/205,496) of images only contained male individuals, while images featuring only female individuals constituted 29.83% (61,230/205,496). The remaining 27.86% (57,251/205,496) featured a mix of male and female individuals. In total, 58.11% (119,413/205,496) of the total images depicted smiling faces. Moreover, 21.81% (44,818/205,496) of individuals wore hats.

To explore how the individuals’ content in the images affects the funding outcome, we built regression models for the attributes. We found the following variables to have a significant impact on funding outcomes. Table 4 presents the outcomes from the regression model.

**Table 4:** The regression results for the attributes of individuals in the image^a^.

Variables	Coefficients	*P* value
Face Count	0.00822	<.001
Female Percent	–0.0201	<.001
Age <18	0.0753	<.001
Age 18-35	0.0432	<.001
Age 36-60	0.0304	<.001
If Smile	0.0446	<.001

^a^The controlled variables included *Funding Amount*, *Donation Count*, *Description Length*, *Title Length*, and *Time*. The significance level is *P*<.001. The *Age* variable takes age ≥60 years as the reference group.

Table 4 includes 4 types of individual variables that have a significant effect on funding outcomes.

- *Face Count*: The results of our experiment indicated a positive correlation between the number of people in the image and funding outcome (*P*<.001).
- *Female Percent*: The regression model indicated that as the percentage of female individuals in the image increases, *Funding Percentage* would decrease. (*P*<.001).
- *Age*: Age ≥60 years was used as the reference group for the regression experiment, which aimed to examine the impact of character age on crowdfunding results. The findings suggested that age-specific personas play a crucial role in determining fundraising ratios, especially among younger crowdfunding initiators (*P*<.001).
- *If Smile*: We found that the presence of someone smiling in the image had a significant positive effect on funding outcome.

### The Impact of Topic on Crowdfunding Outcomes

#### RAM Performance

We used the RAM to analyze image data from 243,795 crowdfunding projects. It obtained tags for various objects in the image and combined them to form an image caption (Table 5).

**Table 5:** Examples of image description^a^.

Image	Tags	Caption
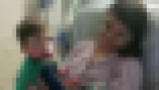	baby|hospital|mother|woman|boy|hospital bed|child|bottle|person|hold|small|young|little	A woman holding a small child in a hospital bed while the baby is being held by a woman who is holding a bottle
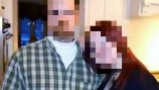	hair|kitchen|woman|shirt|picture|people|glass|man|couple|smile|pose|stand	A man with glasses and a plaid shirt standing next to a woman in a kitchen smiling for a picture

^a^The people in the pictures are mosaicked to protect privacy.

We analyzed the obtained image descriptions using various methods. Word frequency is determined through tag results for statistical analysis. Term frequency–inverse document frequency is a statistical method for evaluating the importance of a word to a document set or to one of the documents in a corpus [49]. Additionally, n-gram is an algorithm based on a statistical language model [50]. As can be seen in Table 6, the most critical words described in the graph in the text analysis obtained from the word frequency, term frequency–inverse document frequency, and n-gram models are all related to people.

**Table 6:** Keywords of the image caption.

Keyword category	Words
Top 10 words with the highest frequency	person, woman, man, picture, people, stand, girl, child, primer, young
Keywords	gadget, control, project picture, maple leave, urinal, organism, crystal, mini, dragonfly, knight
Combination of words	(person, man), (picture, people), (people, person), (person, face)

#### Descriptive Statistics of Image Caption

We used tags to compute word frequencies and generate word cloud visualizations, presenting the variations in content for various types of images. The analyzed image categories comprised differences between images with and without smiles and variances in images depicting individuals of varying age groups.

We categorized the images into “smiling images” and “unsmiling images” based on the presence or absence of smiles on the individuals depicted. The results of the word cloud are shown in Multimedia Appendix 2. We calculated the word frequency and filtered out the 30 words with the highest word frequency in each category of images for comparison. After comparison, we found that the “smiling images” contained more scenes with individuals standing in groups or couples. Conversely, the “unsmiling images” contained more scenes of people lying down in hospital settings.

Images were included in a sample set for each age group if the number of individuals in that age group constituted more than half of the total number of individuals in the image. A comparison of the top 30 words in each category in terms of word frequency revealed that images in the younger than 18 years age group predominantly depict family scenes, while the other age groups tend to depict couples. In addition, hospital scenes are less common in images of people younger than 18 and older than 60 years of age than in the other 2 age groups (Multimedia Appendix 3).

The images differ based on the type of patients with cancer. We selected the 3 cancer type programs with the highest number of occurrences to explore. Corresponding image content for each type of cancer was analyzed with a word cloud, resulting in Multimedia Appendix 4. By comparing the word frequencies of the things that appear in the images for each type of cancer, we found that images related to different types of cancer primarily depict family and medical situations. However, the content varies from project to project. For example, skin cancer projects show a higher frequency of descriptions related to hand images for patients with skin cancer than other projects. Conversely, childhood cancer projects have fewer occurrences of adult features, such as beards, than other projects.

#### Topic Modeling and Regression Models

The use of the DATM resulted in the identification of 7 categories, each consisting of multiple keywords. Our topic coherence score was 0.85 out of 1, indicating strong performance [51]. Our topic diversity score was 0.98 out of 1, indicating that we have effectively covered a wide range of topics [52].

The categories were named based on the commonalities and relationships among the keywords. The names of the topics and their corresponding keywords are listed in Table 7.

**Table 7:** The topic of image caption.

Topic	Related words
Food and cooking	pan, dish, pastry, salad, tea, soup, pie, chocolate, pepper, bread, vegetable
Hair and beauty	hairstyle, earring, braid, beauty, eyebrow, hair, natural, short, lip
City life	garage, luggage, sidewalk, ramp, fire hydrant, cane, building, door, van, train
Clothing and color	white, green, black, blue, red, striped, long, purple, gray, yellow, wear, bald
Rest and play	toy, diaper, crib, bunny, monkey, dog bed, nap, blanket, teddy bear, play with
Medical and health	blood, chest, bandage, thermometer, cord, surgery, dentist, tube, cast, wrist
Diverse locations	rocket, square, site, satellite, knight, traffic light, suburb, mosque, wheat

The regression model shows that the theme of the image has a significant effect on the final fundraising percentage of the project, as shown in Multimedia Appendix 5. We extracted the descriptive words from each image category and organized them into a word cloud for each topic. We analyzed the impact of each theme on the crowdfunding outcome. Red word clouds indicate a positive impact on the crowdfunding outcome, while blue word clouds indicate a negative impact. The word clouds of each topic are ordered from the highest to the lowest degree of influence.

According to Multimedia Appendix 5, several themes have a significant impact on crowdfunding success, each with different levels and directions of influence. In total, 3 specific themes, namely *hair and beauty*, *medicine and health*, and *food and cooking*, have a positive impact on crowdfunding results, in descending order of influence. Conversely, the use of images depicting *city life*, *diverse locations*, and *rest and play* negatively impacted the crowdfunding results for 3 other themes.

### The Impact of Image Features and Image Content on Crowdfunding Outcomes

The word cloud analysis from the previous section displays that images possessing diverse character traits have distinct scenarios and contents. Image content and individual traits exert significant influences on crowdfunding results, respectively. Table 8 illustrates the effects on crowdfunding results when both character traits and image content appear in an image.

The analysis revealed that for certain themes—namely *food and cooking*, *city life*, and *medical and health*—the interaction term *Topic×Face Count* showed a positive and significant coefficient (β values of 0.005, 0.0175, and 0.0118, and *P* values of .008, .007, and .005, respectively), indicating that an increase in the number of faces in these themed images correlates with a higher fundraising proportion.

**Table 8:** The regression results for the individual characteristic image features and the topic of image content in crowdfunding images and topics^a^.

Topic		Topic×Face Count	Topic×Female Percent	Topic×If Smile
**Food and cooking**				
	*r*	0.0050	–0.016	–0.0138
	*P* value	.008	.20	.20
**Hair and beauty**				
	*r*	–0.0051	–0.0381	–0.0043
	*P* value	.06	.50	.80
**City life**				
	*r*	0.0175	0.0177	–0.0441
	*P* value	.007	.80	.002
**Clothing and color**				
	*r*	0.0403	0.035	–0.0919
	*P* value	.40	.20	.005
**Rest and play**				
	*r*	–0.0024	–0.0182	0.0152
	*P* value	.06	<.001	<.001
**Medical and health**				
	*r*	0.0118	–0.0055	0.0004
	*P* value	.005	.90	.20
**Diverse locations**				
	*r*	–0.0046	0.0312	–0.0100
	*P* value	<.001	<.001	.006

^a^The controlled variables were *Funding Amount*, *Donation Count*, *Description Length*, *Title Length*, and *Time*. The significance level is *P*<.001.

Conversely, the *Topic*×*Female Percent* interaction term suggested that a higher percentage of female faces intensifies the negative impact when negative topics are present, particularly in *rest and play* and *medical and health* themes. However, an increase in *Female Percent* within the *diverse locations* theme positively influenced fundraising, as evidenced by a negative coefficient for *Female Percent* (β=–0.0201; *P*<.001) being offset by the positive interaction term *Topic×Female Percent* (β=0.0312; *P*<.001), resulting in a net positive effect.

When considering the presence of a smiley face in conjunction with the image’s topic, the *rest and play* theme correlated with a beneficial increase in funds raised (β=0.0152; *P*<.001). In contrast, themes such as *city life* (β=–0.0441; *P*=.002), *clothing and color* (β=–0.0919; *P*=.005), and *diverse locations* (β=–0.01; *P*=.006) were associated with a negative impact on fundraising.

### Counterfactual Analysis

To better understand the causal relationship between the key variables and crowdfunding success in the above study, we used counterfactual analysis, a widely used method for comparative investigation [53].

In the counterfactual analysis, the indicator of each significant variable is changed to zero, and the predicted values of the model are calculated, comparing the growth of the predicted values before and after the change. Table 9 and Table 10 show the results of the counterfactual analysis. The effect of the variable on the dependent variable as reflected in the results of the counterfactual analysis was consistent with the results of the regression model. For example, *Face Count* (*P*<.001) and *If Smile* (*P<*.001) had a positive causal effect on crowdfunding outcomes and *Female Percent* (*P<*.001) had a negative causal effect.

**Table 9:** Counterfactual analysis of individual characteristic image features and the topic of image content regression^a^.

Variables	Percentage of increase
Face Count	–4.79
Female Percent	2.65
Age <18	–2.62
Age 18-35	–7.16
Age 36-60	–2.90
If Smile	–7.90
Food and cooking	–0.89
Hair and beauty	–0.46
City life	0.43
Rest and play	1.22
Medical and health	–0.28
Diverse locations	0.73

^a^Percentage of increase indicates the percentage increase in the average of the predicted values after changing the data compared to before changing the data.

**Table 10:** Counterfactual analysis of interaction term^a^.

Topic	Topic*×*Face Count	Topic*×*Female Percent	Topic*×*If Smile
Food and cooking	–0.15%	—^b^	—
Hair and beauty	0.09%	—	—
City life	–0.21%	—	0.06%
Clothing and color	—	—	0.03%
Rest and play	0.79%	1.46%	–1.77%
Medical and health	–0.12%	—	—
Diverse locations	2.13%	–1.86%	2.84%

^a^Percentage of increase indicates the percentage increase in the average of the predicted values after changing the data compared to before changing the data.

^b^Not applicable.

## Discussion

### Principal Findings

We found that certain characteristics of individuals in crowdfunding images can significantly influence the success or failure of crowdfunding activities. Specifically, the number of faces, age, gender, and facial expressions, such as smiling, were found to significantly affect the success of crowdfunding campaigns. First, previous studies have shown that the inclusion of faces in crowdfunding campaigns can significantly influence funders’ decisions [38,54]. Specifically, increasing the number of faces used leads to more positive effects. Similarly, our study found that obtaining more characters also leads to more donations. Second, our study suggests that cancer campaigns have better outcomes when a higher percentage of their images feature younger people. This finding is consistent with previous studies [37] suggesting that individuals tend to be more generous in their donations to children when viewing medical crowdfunding programs. Third, several studies [26-28] have demonstrated the positive impact of displaying smiles in crowdfunding projects, and our findings are consistent with this. Such images may contribute to a stronger first impression by increasing viewers’ empathy, which in turn may lead to better crowdfunding outcomes. Fourth, Elitzur and Solodoha [55] found a negative correlation between female leadership and funding success in rewards-based crowdfunding programs. In contrast, Figueroa-Armijos and Berns [56] reported that backers are more likely to support female-led projects than male-led projects. Elitzur and Solodoha [55] and Figueroa-Armijos and Berns [56] argue that the effect of gender on crowdfunding success varies according to the different patterns and stages of project development. However, our study shows that an increase in the proportion of female individuals in the image of medical crowdfunding cancer projects leads to a decrease in crowdfunding success.

For the thematic content of images, we found that images depicting the *hair and beauty*, *medical and health*, and *food and cooking* themes significantly impacted crowdfunding outcomes. The study suggests that these themes may help to awaken empathy and a sense of justice in viewers when viewing such images [57]. For example, beautiful images and photographic narratives related to patient health can evoke hope and empathy in viewers, ultimately encouraging donations [58,59]. However, certain image themes, such as *city life*, *diverse locations*, and *rest and play*, may negatively impact the outcome of a crowdfunding project. These themes are not relevant to medical crowdfunding content and may create additional challenges for readers to understand the primary message, resulting in a negative impression [60,61]. Therefore, a more specific and intentional effort is needed to select images that are relevant to the crowdfunding promoter and help viewers understand the current status of the project. This includes ensuring that images are selected that clearly demonstrate the project’s progress, the promoter’s involvement, and key elements related to the crowdfunding goal. When selecting images, consider the quality, relevance, and expressiveness of the images to ensure that they provide viewers with a comprehensive and clear visual representation of the project.

Our study found that the interaction between individual features in crowdfunding images and image themes in crowdfunding visuals will exert different directional influences on the ultimate fundraising success of medical crowdfunding campaigns. For example, themes such as *medical and health* are positively influenced by an increase in the number of characters in crowdfunding results, while the presence of smiley faces in images for themes such as *city life* is detrimental to crowdfunding results. This dynamic suggests that images with higher thematic and character complexity may deter potential contributors due to the increased cognitive load required to process them, as suggested by Yang et al [16].

The application of counterfactual analysis effectively helps us to distinguish causal effects from statistical correlations [62-64], providing stronger evidence for understanding which factors directly influence the success of crowdfunding projects. In terms of the performance of the results, the characteristics of the 3 components mentioned above, including certain characteristics of individuals, certification image topic, and the interaction, are all consistent with the results of the regression models. This further confirms the causal relationship between these factors and crowdfunding outcomes.

### Practical Implications

Selecting an appropriate image theme that aligns with and emphasizes the informational cues of the crowdfunding project may enhance its persuasive appeal [65] and thereby encourage donation behavior. For example, using *medical and health*–related images with more faces has been shown to increase fundraising effectiveness. This strategy not only increases a campaign’s appeal but also accelerates the achievement of its financial goals.

Analyzing these dynamics can reveal important patterns in crowdfunding results. For example, it can identify which types of projects attract more support or pinpoint image features and themes that are particularly effective in attracting attention and donations. Gathering such insights is critical to refining crowdfunding strategies and developing more effective program designs. These improvements could lead to increased donor engagement and support, thereby improving overall campaign success.

In addition, these findings have practical applications for the design and functionality of crowdfunding platforms. By guiding users to select and upload images that have a higher likelihood of success, such as those depicting younger people or medical scenarios, platforms can strategically influence the visual presentation of campaigns. This targeted approach can optimize the visual impact of these campaigns, potentially increasing their success rates and making the crowdfunding experience more effective for both creators and donors.

For website designers, designers should focus on the selection and presentation of images on the project page to ensure that they are not only clear [66] but also rich in elements that can touch people’s hearts. At the same time, designers can help project sponsors make better choices by creating thematic guidelines or providing a gallery of sample images. Given that different thematic images have different impacts on fundraising results, designers should encourage diversity and provide tools or suggestions to help personalize image content to meet different donor preferences while maintaining overall site consistency and professionalism.

### Limitations and Future Research

There are certain limitations to our research that open the way for potential avenues of future investigation. First, our analysis focused exclusively on crowdfunding campaigns for individual cancer treatments on GoFundMe within the United States. This intentional scope precluded the examination of cancer treatment crowdfunding projects in other countries, leaving room for future studies to explore the dynamics of such campaigns on a global scale.

Second, our study of image content was limited to character features and image descriptions. To increase the comprehensiveness of future research, it is necessary to delve into more complex aspects of image features. This includes a deeper exploration of the interactive actions depicted in the images to provide a more nuanced understanding of how characters visually engage with each other.

Third, the study’s conclusions are based on currently observed image features but do not sufficiently take into account the dynamic changes in audience preferences. Our study is limited to the analysis of image features, while the deeper psychology of the viewer is not sufficiently explored. There is a lack of in-depth analysis of core factors such as emotion and attention.

Fourth, this study focuses on exploring image associations using a quantitative approach, and future research will combine qualitative methods and take advantage of mixed methods research to explore deeper factors of crowdfunding success.

By addressing these limitations and broadening the scope of image analysis, future research efforts can contribute to a more comprehensive and insightful understanding of crowdfunding dynamics in the context of individual cancer treatments.

### Conclusions

In summary, our research examined the influence of image content on the effectiveness of medical crowdfunding initiatives by focusing on specific image features and themes. We rigorously tested 3 hypotheses. Our findings indicate that the representation of individuals in crowdfunding images significantly affects fundraising performance. Certain aspects, such as the presence of multiple individuals, a higher proportion of younger people, and smiles, contribute positively, while a higher proportion of female individuals correlates with a negative impact. The thematic content of the images is also critical, with themes related to *hair and beauty*, *medicine and health*, and *food and cooking* showing positive effects on fundraising. Conversely, themes associated with *city life*, *diverse locations*, and *rest and play* tend to have a negative impact on fundraising efforts. In addition, there is a significant interaction between image themes and the characteristics of the people depicted, influencing the overall fundraising success of the project. This impact varies, increasing or decreasing with the intensity of the image characteristics across themes. Although this study highlights the significant impact of image features on crowdfunding outcomes, viewers' perceptions of images are constantly evolving. Further research into the underlying mechanisms that drive attention in crowdfunding is warranted.

## Appendix

Multimedia Appendix 1 Recognize Anything Model (RAM).

Multimedia Appendix 2 Word cloud of the smile of individuals.

Multimedia Appendix 3 Word cloud of the age of individuals.

Multimedia Appendix 4 Word cloud of the cancer type.

Multimedia Appendix 5 The impact of topic on funding percentage. This figure demonstrates how different image themes affect the funding outcome. The independent variables include the Funding Amount, Donation Count, Description Length, Title Length, and Time. The significance level is *P*<.001. The correlation coefficient (r) denotes the relationship between the image topic and Funding Percentage.

## Abbreviations

DATM: discourse atomic topic modeling
RAM: Recognize Anything Model
