# Supplementary material for: Impact of Image Content on Medical Crowdfunding Success: A Machine Learning Approach
Source: J Med Internet Res. 2024 Nov 15;26:e58617. doi: 10.2196/58617 (PMC11607550; doi:10.2196/58617)

The RAM architecture is divided into three components: the Image Encoder, which employs a Swin Transformer to extract features from images; the Image-Tag Recognition Decoder, which processes image and label features to produce image tags; and the Image-Tag-Text Encoder-Decoder, which takes image features and tags to generate descriptive image captions.

Figure Illustration of RAM's model architecture

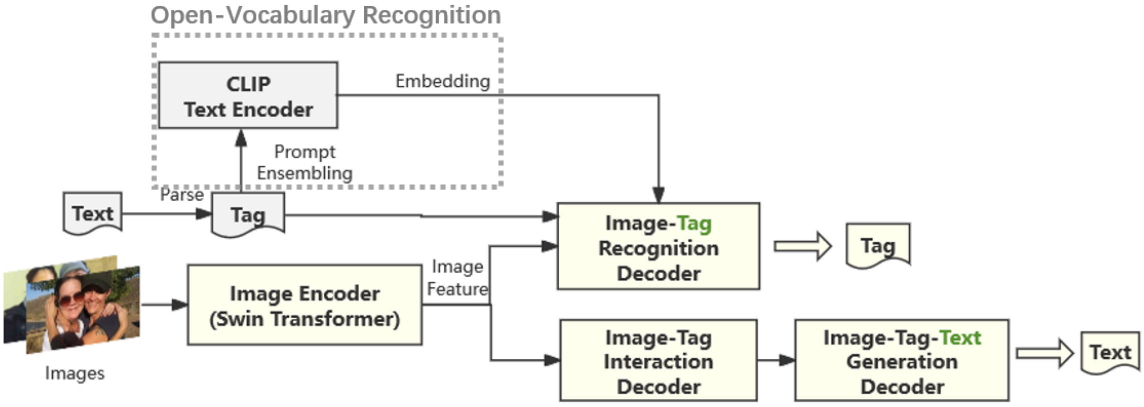

Supplement: Multimedia Appendix 1 [file jmir_v26i1e58617_app1.pdf]
