# Supplementary figures and images for: Impact of Image Content on Medical Crowdfunding Success: A Machine Learning Approach
Source: J Med Internet Res. 2024 Nov 15;26:e58617. doi: 10.2196/58617 (PMC11607550; doi:10.2196/58617)

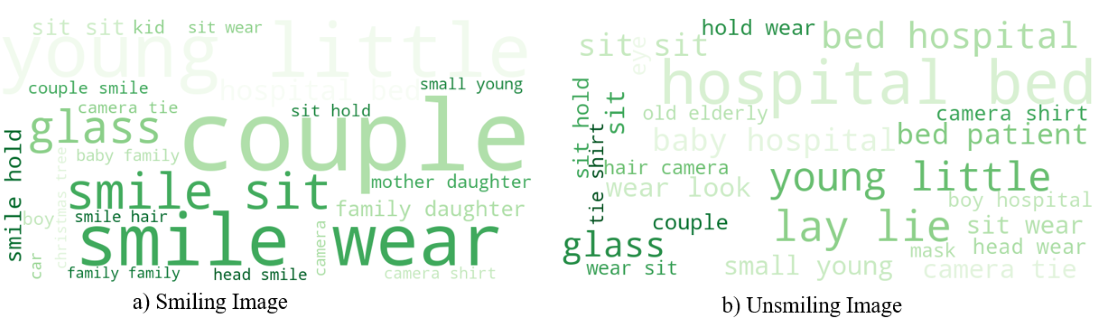

Supplement: Multimedia Appendix 2 [file jmir_v26i1e58617_app2.png]

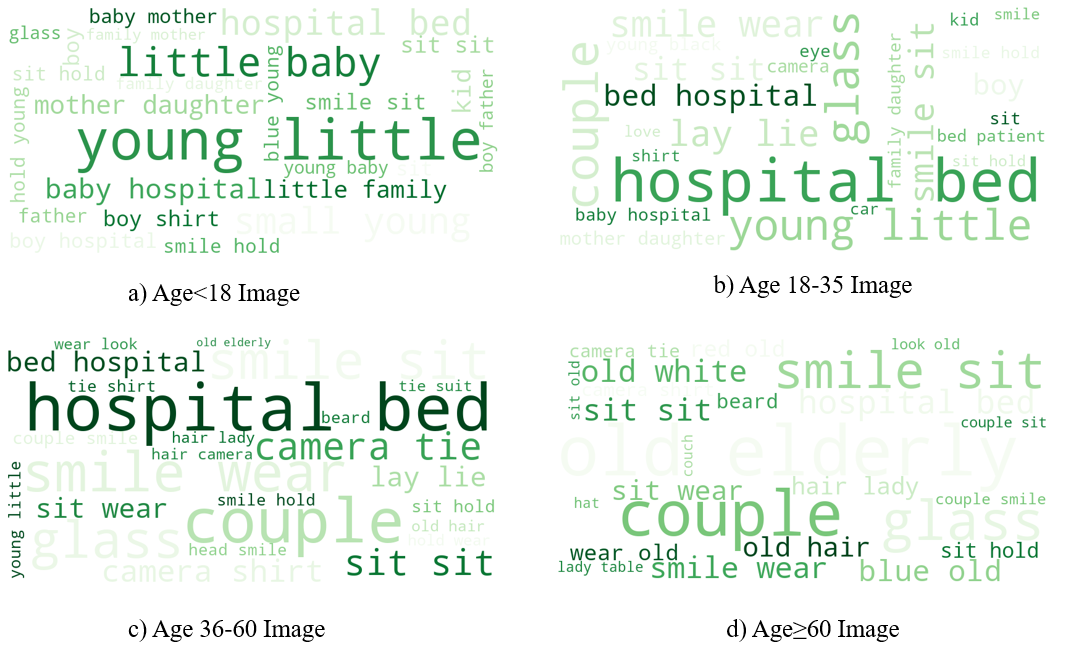

Supplement: Multimedia Appendix 3 [file jmir_v26i1e58617_app3.png]

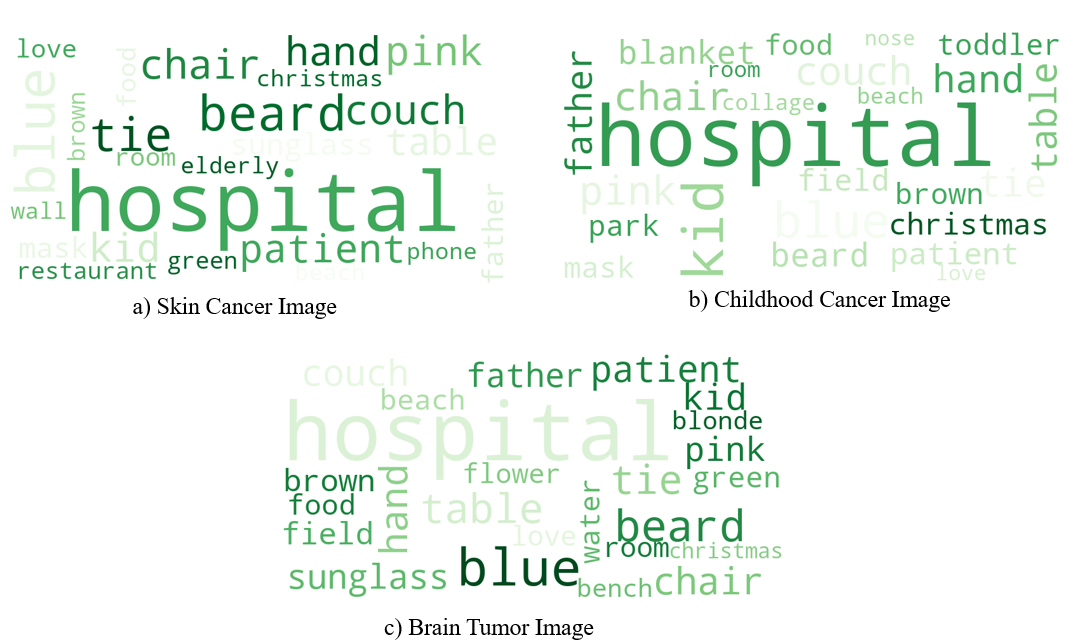

Supplement: Multimedia Appendix 4 [file jmir_v26i1e58617_app4.png]

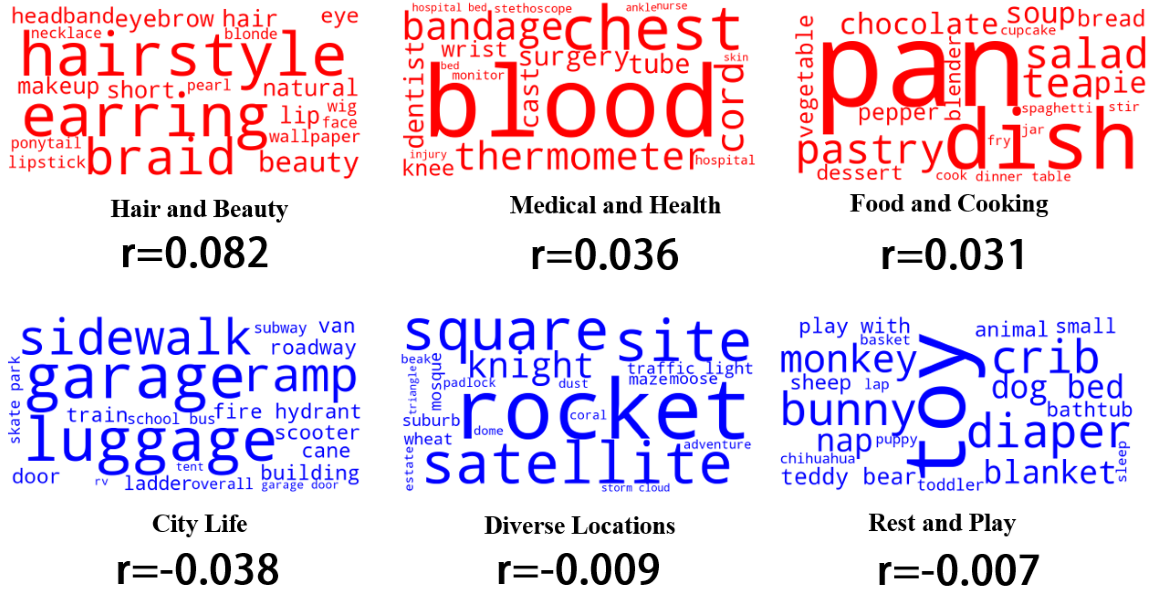

Supplement: Multimedia Appendix 5 [file jmir_v26i1e58617_app5.png]
